# A Systematic Review of Studies Examining Associations between Sleep Characteristics with Dietary Intake and Eating Behaviors during Pregnancy

**DOI:** 10.3390/nu15092166

**Published:** 2023-04-30

**Authors:** Tayla von Ash, Laura Sanapo, Margaret H. Bublitz, Ghada Bourjeily, Amy Salisbury, Sophia Petrillo, Patricia Markham Risica

**Affiliations:** 1 Department of Behavioral and Social Sciences, School of Public Health, Brown University, Providence, RI 02903, USA; 2 Center for Health Promotion and Health Equity, School of Public Health, Brown University, Providence, RI 02903, USA; 3 Department of Medicine, The Miriam Hospital, Alpert Medical School, Providence, RI 02904, USA; 4 Department of Psychiatry and Human Behavior, The Miriam Hospital, Alpert Medical School, Providence, RI 02904, USA; 5 School of Nursing, Virginia Commonwealth University, Richmond, VA 23298, USA; 6 School of Public Health, Brown University, Providence, RI 02903, USA; 7 Department of Epidemiology, School of Public Health, Brown University, Providence, RI 02903, USA

**Keywords:** sleep, diet, energy intake, caloric intake, eating behaviors, pregnancy

## Abstract

Little is known about the association between sleep and diet in pregnancy, despite both behaviors impacting maternal and fetal health. We aimed to perform a systematic review of the available literature on associations between sleep characteristics and dietary intake and eating behaviors during pregnancy, reporting on both maternal and fetal outcomes. We followed the 2020 Preferred Reporting Items for Systematic Reviews and Meta-Analyses (PRISMA) guidelines and conducted our search on 27 May 2021 in the PubMed, EMBASE, and CINAHL databases. The search yielded 6785 unique articles, of which 25 met our eligibility criteria. The studies, mostly observational, published 1993–2021, include data from 168,665 participants. Studies included examinations of associations between various maternal sleep measures with a diverse set of diet-related measures, including energy or nutrient intake (N = 12), dietary patterns (N = 9), and eating behaviors (N = 11). Associations of maternal exposures with fetal/infant outcomes were also examined (N = 5). We observed considerable heterogeneity across studies precluding our ability to perform a meta-analysis or form strong conclusions; however, several studies did report significant findings. Results from this systematic review demonstrate the need for consistency in methods across studies to better understand relationships between diet and sleep characteristics during pregnancy.

## 1. Introduction

Sleep and diet are two important health behaviors that undergo changes during pregnancy and have implications for maternal and fetal health outcomes. Pregnant women report significant changes in their sleep over gestation including a reduction in sleep duration [1,2] and degradation of sleep quality [3], increase in wake after sleep onset, decrease in sleep efficiency, and a higher number of naps [4]. Sleep disturbances, including short sleep duration and poor sleep quality, have been linked to adverse perinatal outcomes such as a higher risk of gestational diabetes, preterm birth, greater gestational weight gain [5], longer labor, and a higher rate of Cesarean delivery [6].

Clinical sleep disorders have also been associated with adverse pregnancy and fetal outcomes. Specifically, restless leg syndrome has been linked to the development of hypertensive disorders of pregnancy [7]. Sleep-disordered breathing has been repeatedly associated with an increased risk for gestational diabetes [8,9], hypertensive disorders of pregnancy [8,10,11], preterm birth [8,11], severe maternal morbidity, and depressive symptoms [12]. Babies born to mothers with a diagnosis of obstructive sleep apnea are also more likely to be admitted to the intensive care unit, to require airway intubation and resuscitation, and to be diagnosed with a congenital anomaly [12,13]. Insomnia has been linked to severe maternal morbidity [14]. Disrupted circadian rhythm and blue light exposure are associated with gestational diabetes or abnormal glucose metabolism [15], and preterm birth [16].

During pregnancy, nutritional needs also demonstrate significant changes and increase to promote fetal growth and development, as well as maternal physiological adaptations of pregnancy (i.e., accumulation of fat reserves) [17,18]. Review of guidelines published by leading international organizations identifies marked consistency in the recommendation for extra calories and protein, especially during the second and third trimesters [18]. Limiting saturated fats, adequate amounts of long-chain fatty acids and possible supplementation with omega-3 fatty acids are also recommended [18]. Reduced consumption of simple sugars, particularly in the form of sugar-sweetened beverages, is recommended to limit excess calories [17].

Supplementation may be needed for adequate intake of iron, folate, Vitamin D, choline, calcium and iodine due to the importance of these in fetal development and the potential for insufficient intake via diet alone [17]. According to the WHO, over 38% of pregnancies are affected by anemia, indicating insufficient micronutrient consumption or absorption over gestation. Anemia, particularly in the first trimester, increases the risk for preterm birth [19] and low birth weight, as well as maternal and perinatal mortality [20]. Insufficient intake of iron and calcium in 91% and 55% of studies, respectively, and excess intake of dietary fat in 55% of studies was found in a meta-analysis of 18 studies of dietary intake pre-conceptually and perinatally [21]. A Cochrane review found that supplementation with iron and folic acid seems to have positive impacts on fetal growth and birth weight including lower small for gestational age (SGA) and preterm birth [22]. Limiting intake of caffeine during pregnancy to 300 mg/day is also recommended [17,18] according to a systematic review [23].

Beyond specific nutrients and supplements, dietary guidelines also recommend healthy diet in the form of diet quality. Poor diet quality is associated with an increased risk for obesity, gestational diabetes, hypertensive disorders of pregnancy, preterm delivery, congenital infections, abnormal fetal growth, and childhood obesity [19,24,25,26,27]. National and international guidelines provide recommendations about appropriate weight gain and healthy nutrition in pregnancy [28,29,30,31]. Consensus across dietary guidelines recommends high-quality dietary intake during pregnancy including consumption of nutrient-rich food groups, managing weight gain, and achieving adequate fluid intake. Specific dietary patterns, such as the Mediterranean Diet, the Dietary Approach to Stop Hypertension (DASH) and others have been studied during pregnancy [17,32,33,34]. The Mediterranean Diet, characterized by high intake of fruits, vegetables and olive oil, with moderate protein intake, has been associated with improvements in risk factors for metabolic syndrome [35] and cardiovascular disease and type 2 diabetes [36] among the general population, and with reduction in unhealthy pregnancy conditions such as gestational diabetes, childbirth complications, low birth weight and prematurity as well as better fetal growth [37].

There is evidence that sleep and diet may affect one another, yet the bulk of the existing literature originates in non-pregnant populations. For example, carbohydrates have been reported to decrease sleep onset latency, and consumption of dairy sources have been reported to increase sleep duration among athletes [38]. Conversely, caffeine increases sleep onset latency, and reduces sleep duration and sleep quality [39]. The timing of meals and meal quantity may affect circadian rhythm, where large portions of food later in the day have the potential to negatively impact sleep [40]. On the other hand, sleep is a pivotal modulator of metabolism and has been argued as a key therapeutic target in metabolic disorders such as obesity [41]. Indeed, in a systematic review, partial sleep deprivation was found to be associated with a positive energy balance (due to an increase in energy intake and no change in energy expenditure), a precursor of excessive weight gain and the development of obesity [42].

Despite the established bidirectional association between sleep and diet, and importance of both sleep and diet on maternal and fetal health, little is known about the associations between sleep and diet in pregnancy. A systematic review by Pauley et al. (2023) demonstrated the infancy of this line of research, identifying only three studies that examined an association between sleep and eating behavior [43]. The aim of this study was to perform a systematic review of the available literature on associations between sleep characteristics and dietary intake and eating behaviors during pregnancy. This study utilizes a comprehensive search strategy and reports on both maternal and fetal outcomes.

## 2. Methods

### 2.1. Protocol and Registration

The present review was conducted in accordance with the 2020 Preferred Reporting Items for Systematic Reviews and Meta-Analyses (PRISMA) guidelines [44]. We prospectively registered the study protocol in the PROSPERO database (CRD42021259982) prior to initiation of literature searches or data collection.

### 2.2. Search Strategy

With assistance from a research librarian, we created individually tailored search strategies for the PubMed, EMBASE, and CINAHL databases to capture studies examining associations between sleep- and diet-related variables during pregnancy. Each search strategy consisted of a search string for the concepts of pregnancy, sleep, and diet. We included search terms related to both nutrition and eating behaviors in the diet search string. The exclusion of animal studies was built into each search strategy as was an English language search limit. No date search limits were applied, and we ran the search on 27 May 2021. The full search strategy is available as Appendix A.

### 2.3. Screening and Eligibility Criteria

The search yielded 9139 hits, which were imported into Covidence, an online systematic review management system, for screening. After removing duplicates, we were left with 6785 unique articles to screen. We screened the titles and abstracts to identify potential studies for inclusion; articles deemed irrelevant were not reviewed at the full-text level. All authors contributed to screening at each level. At each level, articles were independently screened by two individuals. Conflicts were resolved by a third reviewer.

During the full-text screening, we assessed whether each article met eligibility criteria. Eligibility criteria in four areas were included: article type, population of interest, variables of interest, and relationship of interest. To meet eligibility criteria, articles needed to (1) present original research with quantitative outcomes; (2) be published in English in a peer-reviewed journal; and (3) include at least one sleep-related variable and one diet-related variable, at least one of which was a maternal measure during pregnancy. Subsequent variables could include infant measures as well as those during the postpartum period. Finally, (4) the analysis needed to include an examination of the association between at least one sleep- and one diet-related variable.

### 2.4. Data Extraction

All authors participated in data extraction, with two authors independently extracting data from each article in the final pool of studies. We extracted data related to study design, sample characteristics, measures for the variables of interest, and analyses and results related to the relationship of interest, and rated the Melnyk level of evidence for each article based on the analytical approach for the relationship of interest [45]. For example, if the analysis was conducted within the context of a prospective cohort study, but was cross-sectional in nature, the article’s level of evidence was rated as a 4 as opposed to a 3.

## 3. Results

**Figure 1:**
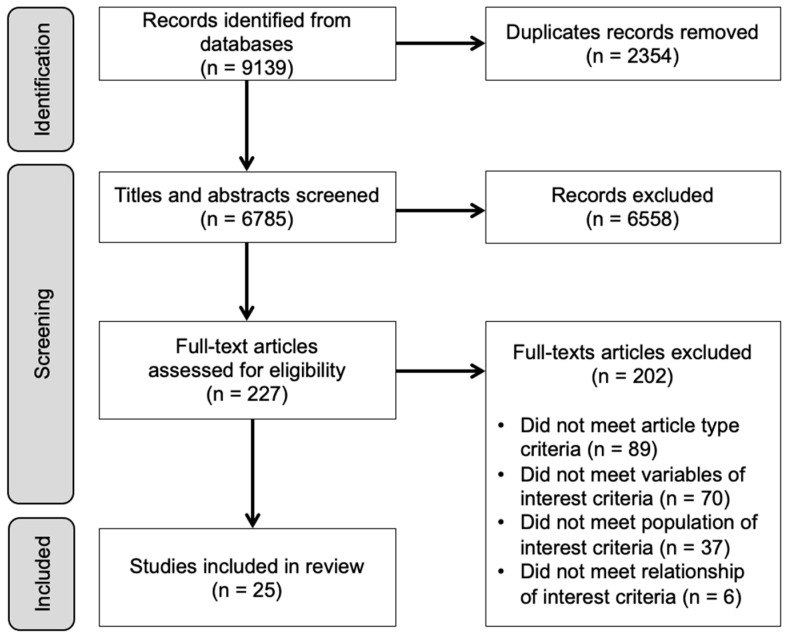
PRISMA flow diagram.

We screened 6785 unique articles. Figure 1 (Prisma Flow Diagram) outlines the selection process which resulted in 25 studies included in the analysis, corresponding to 168,665 pregnant subjects. The characteristics of the 25 studies are presented in Table 1 (organized by year and then alphabetically). They were published between 1993 and 2021 and conducted on five different continents; 10 were conducted in the United States, 7 in Asia, 3 in Europe, 3 in South America and 1 in Australia). Two were randomized controlled trials [46,47] in which pregnant women with overweight or obesity were randomized to lifestyle interventions aimed to reduce gestational weight gain. The level of evidence varied among studies. Twenty-three were observational studies [48,49,50,51,52,53,54,55,56,57,58,59,60,61,62,63,64,65,66,67,68,69,70], which included fourteen that were level four (cross-sectional) [48,49,50,52,53,54,55,57,58,60,61,64,66,67,68,69], and eight that were level three (cohort studies) [51,52,56,59,62,63,65,70]. Only two were level two (experimental studies) [5,47]. Among the observational studies, 19 (83%) were based on relatively low-risk pregnancies [49,51,52,53,54,55,57,58,60,61,62,63,64,65,66,67,68,69,70], while six (25%) enrolled a special population: pregnant individuals with overweight or obese [5,47], overweight or obesity and low-income [48,56], low BMI and low-income [50], or history of major depression [59].

A diverse set of sleep and diet-related measures were used across studies, and the research aims varied in whether the primary outcome(s) of interest were sleep- or diet-related. Thirteen studies framed diet as the exposure and sleep as the outcome [46,50,51,53,54,55,60,61,62,64,66,67,68]; six studies framed sleep as the exposure and diet as the outcome [46,49,50,64,66,70], and two studies examined the associations both ways [58,59], while four studies did not specify [48,53,57,60]. Study results are presented in Table 2, Table 3, Table 4 and Table 5 based on the type of association examined: maternal sleep with energy or nutrient intake (Table 2); maternal sleep with dietary patterns (Table 3); maternal sleep with eating behaviors (Table 4); and maternal exposures (diet or sleep) with fetal/infant outcomes (diet/feeding or sleep) (Table 5). Studies investigating more than one type of association are included in multiple tables. Descriptions of the study measures, which include a variety of objective and subjective measures, are also specified in Table 2, Table 3, Table 4 and Table 5.

Measurement of maternal and infant sleep differed widely among the studies reviewed. Four studies assessed maternal sleep with actigraphy [46,47,56,59], while 19 utilized questionnaire items [48,49,50,53,54,55,57,58,60,61,62,63,64,65,66,67,68,69,70]. For fetal and infant sleep, two studies used direct observation with ultrasound or video recording during feeding [51,52]. For studies that administered questionnaires to mothers, the Pittsburgh Sleep Quality Index (PSQI) was commonly used (n = 8) [48,50,54,57,66,67,69,70], while six studies used single items [53,55,60,61,65,66].

Most studies also used questionnaires to assess diet, only two of which used single questionnaire items [52,61]. Six studies used 24 h dietary recall [53,54,58,60,66,67]. Two studies used standard food frequency questionnaires [49,57], and one a qualitative food frequency questionnaire [69], but protocols varied across studies.

### 3.1. Maternal Sleep with Energy or Nutrient Intake (N = 12)

Twelve studies investigated the association between maternal dietary intake or quality and sleep (Table 2) [5,47,49,50,52,53,54,56,60,66,67,68], seven of which assessed the relationship between total caloric/energy intake and various sleep outcomes [5,49,53,54,56,60,66].

### 3.2. Sleep Variables and Energy Intake

The GUSTO study measured diet with a single 24 h recall during the second trimester among 497 participants of the mother-offspring cohort study in Singapore [66] and the Peking University Birth Cohort measured diet with two non-consecutive 24 h recalls during the first trimester among 4352 participants [54]; neither found an association between energy intake and sleep quality. No association was found between energy intake, calculated based on metabolic parameters and weight (not dietary assessment), with awakenings or sleep duration among 31 overweight or obese participants [46]. Likewise, no association was found between energy intake assessed with two non-consecutive 24 h recalls and nighttime sleep duration among the 3692 participants of the same Peking University Birth Cohort mentioned above [54], and no association was found between energy intake, assessed using a single 24 h recall and nighttime sleep duration or bedtime among 1152 participants of the GUSTO study [60], also mentioned above.

Energy intake among 52 pregnant women with obesity, assessed in the first and third trimesters using energy expenditure (doubly labeled water) and fat deposition (changes in fat density), was not associated with total sleep time [56]. However, women who increased their time in bed were found to have lower energy intake across pregnancy compared to those who decreased their time in bed. Finally, a study of 437 participants in the Australian Longitudinal Study on Women’s Health (ALSWH) found that energy intake, assessed at any time during pregnancy using a food frequency questionnaire, was associated with sleeping behavior patterns that were identified via latent class analysis in crude models [49]. Women with average sleep duration and adverse sleep symptoms had higher energy intake compared to those with average sleep duration, but no adverse sleep-related symptoms [49]. These findings did not persist after adjustment for potential confounders [49].

An intervention study providing meal replacement for two meals per day aimed to restrict caloric intake to promote weight control and adherence to guideline recommendations of gestational weight gain [47]. No difference was found in actigraphy-measured sleep duration between the intervention and usual care comparison group [47].

### 3.3. Sleep Variables and Nutrient Intake

The relationship between nutrient intake and various aspects of sleep was also examined. Sleep and dietary fat were assessed by two studies [49,50]. In the first study, wherein 213 pregnant participants were recruited at Special Supplement Program for Women Infants and Children (WIC) sites in Michigan and assessed at a single time point, which included 36% in the first trimester, 32% in the second trimester and 32% in the third trimester. Dietary fat intake, measured by the Block screener was found to be directly associated with nighttime sleep disturbance in the overall cohort (*p* < 0.01) [50]. However, path analysis examining participants by trimester demonstrated that an association of dietary fat with depression (*p* < 0.05), and not nighttime sleep disturbance, was found during the first trimester [50]. That same study showed a path connecting sleep latency to dietary fat through the association with both fruit and vegetable intake (*p* < 0.05 for each leg of the path). Among second-trimester participants, an association of dietary fat with nighttime sleep disturbances (*p* < 0.01) and with fruit and vegetable intake (*p* < 0.05) was found, but none of these factors connected to depression [50]. Third-trimester participants were found to have an association between dietary fat and nighttime sleep disturbances (*p* < 0.05), which was also associated with depression (*p* < 0.01). An association of monounsaturated fat intake, but not overall fat intake, with sleep patterns was identified, as described above among the ALSWH sample [49]. Women with average sleep who also had adverse sleep symptoms consumed less monounsaturated fat than women with average sleep and no adverse sleep symptoms (*p* < 0.05) [49].

The relationship between carbohydrate intake and sleep was also assessed in two studies [49,67]. In cross-sectional analyses, the percentage of energy consumed as starch was found to be significantly higher among women with average sleep who also had adverse sleep symptoms, in comparison to women with average sleep and no adverse sleep symptoms (*p* < 0.05), though no differences in overall carbohydrate intake or sugar consumption were found between sleep pattern groups [49]. However, in multivariate models, overall carbohydrate intake was higher among women with average sleep who also had adverse sleep symptoms, in comparison to women with average sleep and no adverse sleep symptoms (*p* < 0.05), though no relationship was found for starch or sugar consumption with sleep patterns [49]. In the second study [67], intake of sugar-sweetened beverage, assessed by three 24 h recalls, was associated with short sleep duration and poor sleep quality, even after adjustment for potentially confounding variables. However, this cross-sectional study did not separate sugar-sweetened beverages that did or did not contain caffeine.

Additionally, the GUSTO mother-offspring cohort study in Singapore assessed discretionary calories as the sum of energy from caloric beverages (≥5 kcal; excluding plain water, diet soda, and unsweetened coffee, tea, and cow’s milk), local cakes, desserts, and snacks based on 24 h recall data. No association was found between discretionary calories with either sleep quality or duration assessed using the PSQI [66].

Caffeine consumption was assessed with maternal diet in two studies [52,68]. Maternal caffeine consumption during pregnancy was assessed with obstetrical outcomes among 750 participants recruited during the second trimester [52]. They reported that caffeine consumption during the second and third trimesters was associated with higher maternal depression and anxiety scores, and more cigarette use. Controlling for these variables, the investigators found that higher maternal caffeine consumption was significantly related to lower maternal sleep effectiveness as assessed by a Sleep Scale [52]. However, a study of 266 pregnant women in Poland late in pregnancy found no difference in insomnia between participants who did and did not abstain from coffee consumption during pregnancy [68].

### 3.4. Maternal Sleep with Dietary Patterns (N = 9 Studies)

Dietary intake was also assessed as food group consumption or dietary patterns (Table 3) [50,53,54,55,56,57,58,66,69]. Fruit and vegetable intake among Michigan WIC participants, assessed using a rapid food screener, was associated with sleep latency in the first and third trimester, but not in the second, and no association of fruit and vegetable intake with sleep quality or duration was found in any trimester [50]. In contrast, others assessing US respondents to the Behavioral Risk Factor Surveillance System (BRFSS) found that fruit and vegetable consumption as well as fruit consumption alone were associated with increased odds of meeting or exceeding sleep duration recommendations, but this association was only found when assessing sleep as an ordinal variable, while no association was found in linear models [55]. Several studies also examined associations between sleep and diet quality. Healthy Eating Index was calculated for two studies using very different dietary data collection. Diet was measured among pregnant women with obesity using food photography via the SmartIntake app collected in early (13–17 weeks’ gestation) and late (35–37 weeks’ gestation) pregnancy [56], whereas diet among women of any weight status was assessed using a single 24 h recall at 24–28 weeks’ gestation [66]. The first study using SmartIntake found no significant association between diet quality and time spent in bed or total sleep time [56]. The second that used 24 h recall also did not find an association between diet quality and sleep duration but did find a slightly higher HEI score (higher quality diet) among participants with good quality sleep (54.6) compared with those with poor quality sleep (52.0), though that association was no longer statistically significant when controlling for anxiety [66]. Chronotype (a person’s circadian preference in behavioral and biological rhythms relative to the external light–dark cycle) in the first trimester was associated with HEI scores calculated from three 24 h recalls such that higher whole grain and lower fruit HEI sub-scores were associated with higher HEI scores, though no difference in overall HEI score by chronotype was identified [58].

Four studies examined associations between various measures of sleep and several different types of dietary patterns [53,54,57,69]. A Mediterranean food pattern, assessed with a food frequency questionnaire among Spanish women at 11–13 weeks and 34 weeks’ gestation, was associated at both time periods with better sleep quality, assessed using the Spanish PSQI. The investigators also examined individual items of the Mediterranean diet, which showed that higher olive oil consumption at both time periods, and fruit early in pregnancy were associated with better sleep quality, while consumption of red meat and sub-products late in pregnancy was associated with lower sleep quality. A prospective study among pregnant Chinese women examined cross-sectional dietary intake and sleep early in pregnancy [53,54]. Dietary intake was measured with two days of 24 h recall from which total caloric intake was calculated, as described previously. However, the authors also categorized participants’ diets as balanced, more meat, veggie-rich, or vegan, though no methods for determining these categories were provided. Based on these non-specific methods, participants described as having a vegetarian or more-vegetables diet type were more likely to have poor sleep quality, as measured by the PSQI, than those with a balanced diet [54]. Additionally, those consuming a vegan diet were more likely to have either short or long sleep duration, which were self-reported. Dietary patterns of pregnant participants during their first trimester of gestation in the Chinese Pregnant Women Cohort Study [69] were assessed by principal components analysis of data derived from a 17-food group-based food frequency questionnaire. These analyses revealed five patterns: plant-based, vitamin-rich, animal protein-rich, bean products, and high fat, which were described by quartiles. Plant-based and vitamin-rich dietary patterns were associated with less sleep disturbance, and a high-fat dietary pattern was associated with greater sleep disturbance [69].

### 3.5. Maternal Sleep with Eating Behaviors (N = 11 Studies)

Eleven studies investigated the association between variables related to maternal eating behaviors with sleep (Table 4) [48,56,58,60,61,64,65,66,68,70], six of which included a measure related to nighttime eating or fasting [48,58,60,61,66,68]. Among African American women assessed between 14- and 24-weeks’ gestation, night eating was associated with lower sleep duration and quality, and higher sleep onset latency assessed using questions from the PSQI [48]. Among 673 s-trimester pregnant women in Singapore, night-eating was associated with a later bedtime, but did not find an association between the number of eating episodes with sleep duration, as reported in response to a single question [60]. The GUSTO mother-offspring cohort study in Singapore found no association between nighttime eating, nighttime fasting interval, and frequency of consumption episodes with either sleep quality or duration assessed using the PSQI [66]. Relatedly, an early pregnancy cross-sectional study in Brazil did not find an association between sleep chronotype and nightly fasting, eating duration, time of first or last meal, and number of meals [58].

Two studies specifically considered the association between nighttime eating and insomnia [61,68]. A study of third-trimester pregnant women in Poland found that women reporting insomnia, using the Athens Insomnia Scale, were more likely to eat at night [68]. Another study of pregnant women in Vietnam from 12 to 33 weeks’ gestation found that short meal-to-bed time, based on self-reported times, was a risk factor for reflux-related insomnia [61].

Four studies assessed eating behaviors including binge eating and food cravings [48,56,64,70]. One study of African American women with overweight or obesity between 14- and 24-weeks’ gestation found that sleep latency and quality (assessed with PSQI) were associated with overeating episodes, and poor sleep quality was associated with binge eating episodes. However, no associations were found between sleep duration and dietary restraint, overeating, or binge eating episodes [48]. The Norwegian Mother and Child Cohort Study (MoBa), which is a large, prospective population-based cohort study, assessed mothers with binge eating disorder (BED) symptoms and their associations with sleep, which was self-reported as sleep problem occurrence and during which weeks of gestation, sleep duration and satisfaction [65]. Participants reported BED symptoms before and during pregnancy. Participants reporting pre-pregnancy BED that remitted during pregnancy, or those reporting incident BED during pregnancy, were more likely to report sleep problems, and the likelihood of those problems increased with each trimester [65]. Incident BED during pregnancy was associated with both long and short sleep durations, whereas BED symptoms before or during pregnancy, or pre-pregnancy BED that remitted during pregnancy was not associated with long or short sleep. All three BED groups reported higher odds of sleep dissatisfaction than participants without any BED symptoms or history [65].

Food cravings were also examined with respect to sleep in two studies. One study of 52 pregnant women with obesity assessed sleep with wrist-worn actigraphy over six consecutive nights during early and late pregnancy. Pregnant women in this study who increased time in bed from early to late pregnancy also reported an increase in food cravings during that time compared to women who decreased time in bed or those who had no change in time in bed [56]. Another study of 245 pregnant women in Brazil assessed chronotype via mid-sleep time on free days with correction for calculated sleep debt across various times in pregnancy. Participants with sleep chronotype of evening types, which have ≥5 h difference between weekend and weekday average sleep duration, were more likely to report relief from negative states and feelings as a result of eating (as measured by the Food Craving Questionnaire-Trait) than those in non-evening chronotype (<5 h difference between weekend and week day average sleep duration). Compared to participants reporting morning type (<3.59 h difference between weekend and weekday average sleep duration), those with evening type reported both anticipation of relief from negative states and feelings as a result of eating, anticipation of positive reinforcement that may result from eating. Intense desire to eat was more likely to be found among evening type compared with morning type participants, whereas both intense desire to eat, and anticipation of positive reinforcement that may result from eating were found between evening type and non-evening type participants [64]. Additionally, The Pregnancy Eating Attributes Study (PEAS) followed 373 women from early pregnancy to one year postpartum [70]. Eating behaviors measured included cravings, which were assessed using questions developed by the authors wherein participants listed cravings and rated their strength; hedonic hunger, based on the Power of Food Scale; and addictive-like eating based on a modified Yale Food Addiction Scale. Sleep quality, assessed with the PSQI, was associated during pregnancy with cravings frequency and strength, but not with hedonic hunger or addictive-like eating. Additionally, change in sleep quality from pregnancy to post-partum was not associated with changes in hedonic hunger or changes in addictive-like eating [70].

### 3.6. Maternal Exposures with Fetal/Infant Outcomes (N = 5 Studies)

Three studies [51,52,62] examined the associations between maternal caffeine consumption during pregnancy on fetal and/or infant sleep (Table 4) [51,52,62]. The impact of high vs. low maternal caffeine consumption (reported use at study entry of >500mg (N = 10) vs. <200 mg (N = 10), respectively), on fetal behavioral states was assessed throughout the third trimester (30 to 40 weeks of gestational age) [51]. The fetuses of women who reported high caffeine consumption spent less time in active sleep and more time in arousal than fetuses of women with low caffeine consumption [52]. Caffeine consumption during the second and third trimesters was associated with higher maternal depression and anxiety scores, more cigarette use, and lower infant birth weight. Controlling these variables, higher maternal caffeine consumption was significantly related to higher amounts of infant active sleep, and more stress signs, and that caffeine consumption was correlated with lower maternal sleep effectiveness during the first 24 h after delivery [52]. In contrast, the last did not find any significant associations between maternal caffeine consumption during late pregnancy and infant sleep at 3 months of age [62].

One study [63] examined the association between maternal fermented food intake during pregnancy and infant sleep. The investigators found that intake of fermented food, especially miso, during the second and third trimesters, was associated with less infant sleep at one year of age [63].

Finally, another study examined the impact of a maternal sleep-related exposure on an infant diet-related outcome [59]. The investigators found that low maternal sleep efficiency in the third trimester was associated with lower likelihood to initiate breastfeeding, with a trend for a similar association between sleep efficiency and feeding status at 16 weeks postpartum.

## 4. Discussion

In this systematic review, we observed significant heterogeneity in studies examining associations between maternal sleep and diet. Differences in existing studies were observed in the selection of exposures and outcomes (sleep vs. diet) and their definitions. Participant samples range in numbers from less than 100 to over 17,000. Pregnant individuals were studied either during a specific trimester, or at any time during pregnancy. Samples also varied in terms of racial and ethnic representation with some studies including participants with a diverse range of demographics, while others included a more homogenous sample. Studies also varied in specific population of pregnant individuals assessed with some assessing participants with different weight statuses, or only including those with overweight or obesity pre-pregnancy. Further, the methodology used to examine sleep varied, but consisted of subjective questionnaires for most studies. Dietary variables of interest also varied with some studies examining diet quality and intake while others examined specific nutrient intake, food groups, or dietary patterns. This marked heterogeneity in the existing literature has precluded our ability to perform a meta-analysis of the data or even to form strong conclusions. Like findings from the Pauley et al. (2023) [43] review, our findings support the need for more longitudinal studies and randomized controlled trials. Notably, only two of the selected studies were designed as a randomized controlled trial. Additionally, a large proportion of data was obtained from low-risk samples, impacting the ability to extrapolate to higher-risk populations.

Energy intake was not found to be strongly associated with sleep among pregnant women. Most (5) of the seven studies that examined energy intake and sleep found no association of caloric intake with sleep quality [54,66] or duration [46,53,60]. The only randomized controlled trial of a partial meal-replacement to limit or control caloric intake also found no differences between intervention and comparison group in actigraphy-measured sleep outcomes [47]. Two studies found an association between energy intake with sleep variables, though methods and conclusions were very dissimilar. Women with obesity who increased their time in bed over the course of pregnancy had lower energy intake compared to those for whom time in bed decreased or stayed the same [56]. In another study, women with average sleep duration and adverse sleep symptoms had higher energy intake compared to those with average sleep duration, but no adverse sleep-related symptoms [49]; however, these findings did not persist in multivariate analysis.

Other studies of non-pregnant adults measuring energy intake and sleep duration have consistently shown that short sleep is associated with higher caloric intake, and that hormonal changes associated with short sleep may be responsible for the small changes in energy intake [71]. The set of papers in our review nicely highlights the wide variations in measurement that included first, second, third, or multiple trimesters. Energy intake was assessed using one or two 24 h recalls, a food frequency questionnaire, and several indirect measures using metabolic estimates of energy usage. Additionally, sleep was assessed in these studies as self-reported and actigraphy-measured sleep duration, self-reported sleep quality, and categories of sleep duration and/or sleep quality. So, though no associations were found between energy intake and sleep in these seven papers, the lack of consistency in methods challenges our ability to draw strong conclusions regarding this relationship.

The relationship between dietary fat intake and sleep among pregnant women is not clear from the two studies that examined this relationship. Neither assessment of sleep (pattern vs. sleep duration, quality, nighttime disturbances, latency) nor diet fat (screener vs. food frequency questionnaire) were assessed consistently across these studies. Higher overall dietary fat intake was associated with more sleep disturbance among Michigan WIC participants [50]. Additionally, women who reported average sleep with adverse sleep symptoms consumed less monounsaturated fat than women with average sleep and no adverse sleep symptoms [49]. Studies among non-pregnant adults of dietary fat with sleep outcomes are not common, but have collectively indicated that higher fat intake is associated with short sleep [71], and insomnia [72], and that saturated fat intake is overall negatively associated with sleep wellness [73]. Little to no literature is available to review the relationship between monounsaturated fat and sleep in other study populations.

Findings regarding the association between carbohydrate intake and sleep were scarce and did not support strong conclusions. The two studies that assessed carbohydrate intake showed higher carbohydrate consumption among women with average sleep, but adverse sleep symptoms, compared to those with average sleep without adverse sleep symptoms [49]. Additionally, higher sugar-sweetened beverage intake was associated with short sleep and poor sleep quality, though caffeine consumption was not taken into account [67]. Consistent association between carbohydrate consumption and sleep outcomes has also been inconclusive in studies of non-pregnant adults [73]. Differentiating between types of carbohydrate may shed light on this issue as intake of a high glycemic index diet or glycemic loads have been posited to be associated with higher risk of insomnia [73,74], which may be due to alterations in amino acid balance or a stimulated inflammatory response [74]. Beyond examining single nutrient diet composition, combinations of nutrients, such as high carbohydrate, low fat, have also been considered as potentially important in the relationship between diet and sleep [75].

Caffeine may be the single most studied substance in this literature. Caffeine consumption in our review was associated with lower maternal sleep effectiveness after controlling for depression and anxiety [52], though abstaining from coffee consumption was not associated with insomnia risk [68]. The impact of caffeine consumption on nighttime sleep is also not clear in non-pregnant adults [76,77], due to the cyclical impact of caffeine on performance enhancement and mitigating sleep deprivation and caffeine withdrawal [76].

A variety of dietary patterns were assessed with sleep in nine papers in this review. Higher fruit and vegetable consumption was inconsistently associated with sleep latency in one small study [50] and broadly associated with meeting or exceeding sleep duration recommendations in a larger, population-based study [55]. Higher fruit and vegetable consumption among non-pregnant adults has been consistently associated with achieving recommended sleep in observational studies, though experimental studies have been inconsistent [78].

Broadly assessing diet quality with sleep, HEI was not consistently found to be associated with sleep duration [56,66], but was associated with sleep quality in one study [66], and HEI sub-scores of higher whole grains and lower fruit consumption were associated with chronotype in another [58]. In non-pregnant adults, HEI has also been found to be associated with various sleep outcomes. HEI assessed using two 24 h recalls was significantly lower among short and long sleepers compared to those reporting the recommended 7–9 h of sleep among nationally representative NHANES study participants [79]. Additionally, among NHANES participants, higher HEI is associated with lower risk of sleep disturbance [80]. Broadly, a review of 29 studies indicates that higher dietary quality is associated with better sleep quality [81], though not all studies used the HEI index and methodological inconsistencies limit the strength of the evidence and preclude causal inference, as we have also found.

Nighttime eating is associated with misaligned circadian rhythms, or “eating jetlag”, which can be associated with positive energy balance and weight gain among shift workers and others who have milder shifts in eating behavior in the general population [84]. The relationship between nighttime eating and insomnia among pregnant women correlates these two nighttime occurrences, but it is unclear if pregnant women who experience insomnia awaken because of hunger, or eat because they were awake [68]. Insomnia has been found to be associated with intake of higher glycemic index (GI) diet in the Women’s Health Initiative Observational Study [74], which has been hypothesized to be mediated by inflammation [73,74]. GI, originally defined as the postprandial glucose response to food, [85] is calculated for and summed over all carbohydrate-containing foods [74].

Other dietary patterns, such as the Mediterranean diet, veggie-rich, meat-based, plant-based, vegetarian, vegan and other descriptors were mixed in association with various sleep outcomes, and inconclusive in our review. Research in non-pregnant populations has found that Mediterranean diet adherence is positively associated with sleep duration and indicators of better sleep quality [82]. Other studies have found changes in eating patterns associated with short sleep [83]. Among 15,199 adult participants of the NHANES from 2005 to 2010, short sleepers were found to eat earlier and later, consume more calories as snacks (than meals) and consume more sugar and caffeine than participants reporting longer sleep [83].

Sleep seems to also play an important role in eating disorders, including BED [86] and BED. For example, a systematic review by da Luz et al. (2023) [87] found that people who binge eat exhibit poorer overall sleep quality compared to people who do not binge eat, and may have more daytime sleepiness, insomnia, and difficulty falling asleep. BED has even been proposed as a possible circadian disorder [88]. More studies of these associations might explain these relationships as well as the findings among pregnant women reviewed here [48,65].

Cravings have been found to be associated with sleep in various non-pregnant populations. Lack of sleep has been found to be associated with cravings among women without obesity [89], and healthy young adults [90]. Food cravings were associated with poor sleep quality among shift-workers [91]. Higher sleep efficiency was found to be associated with lower sweet cravings among adolescents [92]. Chronotype was not associated with food cravings in several studies [93,94,95]. None of these findings are consistent with the associations of time in bed [56] and chronotype [64] with cravings found among pregnant women in this review.

Interpretation of these results must be considered with the limitations of the processes and studies themselves. As mentioned above, 60% of the studies had a low level of evidence (less than level 4) and only two of the studies were RCTs. Additionally, a wide range of methods and outcome measures were used, which limits the conclusions that can be drawn across studies. Further, most of the studies were conducted only with participants with low-risk pregnancies. However, the review of this research was conducted with systematic processes according to the PRISMA framework, so the literature presented is representative of the state of this research.

## 5. Conclusions

This literature review demonstrated the need for improved consistency or standardization in methods and a relative lack in longitudinal data, but also highlights great opportunities for future research. For example, higher-powered studies are needed to look both separately and across pregnancy trimesters to examine the relationship between maternal diet, sleep, and outcomes throughout the course of pregnancy. Additionally, more rigorous measures are likely needed to assess smaller components of diet such as monounsaturated fat, as day-to-day variations in intake require great precision of measurement; however, this will likely result in more burdensome assessment.

## Figures and Tables

**Table 1:** Study and sample characteristics (n = 25).

Author, Year, Country	Study Design, Melnyk Level of Evidence	Sample Size	Maternal Age at Baseline (Mean ± SD or % Distribution)	Time of Recruitment	Parity (Mean ± SD or % Multiparous)	BMI/Weight Status of Sample (Mean BMI ± SD or % Distribution)	Special Population (Yes/No)	Eligibility Criteria
Devoe et al., 1993, USA [51]	cohort, 3	20	high consumers (n = 10): 23.8 + 5.6; low consumers (n = 10): 26.2 + 7.2 (*p* < 0.01)	1st and 2nd trim	high consumers (n = 10): 0.7 ± 0.8; low consumers (n = 10): 1.5 ± 2.4 (*p* < 0.01)	not reported	no	singleton, normal glucose tolerance, no evident maternal or obstetric complications, no exposure to smoking or medications, normal fetal growth and amniotic fluid volumes
Diego et al., 2008, USA [52]	cohort, 3	128	not reported	2nd and 3rd trim	not reported	not reported	no	no pregnancy complications including preeclampsia and gestational diabetes, no HIV or any other infectious disease, no psychotropic medications or recreation drugs during pregnancy
Allison et al., 2012, USA [48]	cross-sectional, 4	120	25.2 + 5.1	2nd trim	68.3% multiparous	32.4 + 7.8	yes (low-SES African American women with a pre-pregnancy BMI ≥ 25 kg/m^2^)	African American, ≥18 years of age, pre-pregnancy BMI of ≥25 kg/m^2^, singleton pregnancy, no preexisting diabetes mellitus, autoimmune disorder, or regular use of steroid treatment
Santos et al., 2012, Brazil [62]	cohort, 3	885	19.3% < 20, 70.8% 20–35, 9.8% > 35	at delivery	55.9% multiparous	not reported	no	singleton pregnancy
Ulman et al., 2012, Norway [65]	cohort, 3	72,435	not reported	2nd trim	44% multiparous	3.2% < 18.5, 66.0% 18.5 < BMI < 25, 21.5% 25 < BMI < 30, 9.3% > 30	no	singleton pregnancy
Chang et al., 2015, USA [50]	cross-sectional, 4	213	1st trim (n = 75): 26.08 + 5.70; 2nd trim (n = 68): 26.07 + 5.38; 3rd trim (n = 68): 25.70 + 5.59 (non-sig.)	any trim	not reported	1st trim (n = 75): 33.60 + 7.09; 2nd trim (n = 68): 31.60 + 4.71; 3rd trim (n = 68): 31.97 + 5.54 (non-sig.)	yes (low-SES with a pre-pregnancy BMI ≤ 25 kg/m^2^)	≥18 years of age, speak and understand English, enrolled in the Special Supplemental Nutrition Program for Women, Infants, and Children (WIC)
Duke et al., 2017, USA [55]	cross-sectional, 4	2951	30% 18–24, 53% 25–34, 17% 35–44	any trim	not reported	not reported	no	pregnant Behavioral Risk Factor Surveillance System (BRFSS) participant, 18–44 years of age
van Lee et al., 2017, Singapore [66]	cross-sectional, 4	497	good sleepers (n = 271): 30.8 + 4.5; poor sleepers (n = 226): 30.7 + 5.0 (non-sig.)	1st trim	good sleepers (n = 271): 56.8% multiparous; poor sleepers (n = 226): 53.1% multiparous (non-sig.)	good sleepers (n = 271): 22.8 + 4.2; poor sleepers (n = 226): 22.5 + 4.5 (non-sig.)	no	part of larger cohort, intention to deliver in one of the two major study hospitals in Singapore, agree to donate placenta, cord, and cord blood at delivery, spouse had homogenous parental background of Chines, Malaysian, or Indian descent, no serious health conditions (e.g., type 1 diabetes, cancer, or psychological disorders), no probable major depression
Wolynczyk-Gmaj et al., 2017, Poland [68]	cross-sectional, 4	266	30.56 ± 4.95	3rd trim	not reported	with insomnia (n = 42): 28.8 (25.2, 31.6); without insomnia (n = 160): 26.4 (24.3, 28.9) (non-sig.)	no	in 3rd trimester
Gontijo et al., 2019, Brazil [58]	cross-sectional, 4	100	27.3 ± 5.7	1st trim	not reported	pre-pregnancy: 24.0 ± 4.4; current: 24.7 + 4.6	no	healthy pregnant woman, singleton, ≥18 yrs of age, had prenatal visit no later than 12 wks gestation, no positive test for HIV, syphilis, toxoplasmosis, rubella, cytomegalovirus, or varicella
Phelan et al., 2018, USA [47]	RCT (behavioral lifestyle intervention with partial meal replacement to reduce GWG), 2	257	30.3 ± 5.42	1st and 2nd trim	72.7% multiparous	32.5 ± 5.3	yes (overweight or obese)	gestational age between 9 and 16 wks at enrollment, BMI ≥ 25, English or Spanish speaking, age ≥ 18 yrs, singleton pregnancy, glycated hemoglobin < 6.5, no major health diseases (e.g., heart disease, cancer, renal disease, diabetes), no current substance abuse, no current treatment for a serious psychological disorder (e.g., schizophrenia, bipolar disorder), no contraindications to aerobic exercise
Bennett et al., 2019, Australia [49]	cross-sectional, 4	437	33.5 ± 1.4	any trim	LC1 (n = 167): median 2 (IQR:1–3); LC2 (n = 173): median 2 (IQR:1–2); LC3 (n = 97): median 2 (IQR:2–3) (*p* < 0.01.)	LC1 (n = 167): median 23.1 (IQR:20.8–26.5); LC2 (n = 173): median 23.5 (IQR:20.8–26.4); LC3 (n = 97): median 24.1 (IQR:21.7–28.2) (non-sig.)	no	pregnant women part of larger cohort, sleep duration between 3 and 12 h, energy intake between 4500 and 20,000 kJ
Loy et al., 2020, Singapore [60]	cross-sectional, 4	673	30.9 ± 5.0	1st trim	not reported	53.2% < 23, 46.8% ≥ 23	no	attended antenatal care in 1st trimester, ≥18 yrs of age, homogenous parental ethnic groups (Chinese, Malay, or Indian), no chemotherapy or psychotropic drugs, no type 1 diabetes, singleton pregnancy, plausible total daily energy intake (500–3500 kcal)
Sugimori et al., 2019, Japan [63]	cohort, 3	72,624	31.5 at delivery	1st and 2nd trim	58.8% multiparous	4.9% < 18.5, 79.6% 18.5-< 25, 15.4% ≥ 25	no	resident in study area, expected to reside continually in Japan for foreseeable future, expected delivery between August 2011 and mid 2014, able to understand Japanese and complete self-administered questionnaires, live singleton birth
Du et al., 2021, China [53]	cross-sectional, 4	3692	median: 29 (IQR: 27–32)	1st and 2nd trim	37% multiparous	median: 22 (IQR: 20–24) (pre-pregnancy)	no	18–45 yrs of age, <14 wks gestation at recruitment, resident of study area with no plans to move, intention to undergo prenatal care and delivery at specific hospital, no cognitive mental disorders, no pre-pregnancy diabetes or hypertension, no cardiovascular, liver, kidney, or autoimmune disease, singleton pregnancy
Flanagan et al., 2021, USA [56]	cohort, 3	52	27.4 ± 0.7	1st and 2nd trim	52% multiparous	36.3 ± 0.7	yes: obese	18–40 yrs of age, BMI ≥ 30 kg/m^2^ at recruitment, no smoking, no alcohol or drug use, no hypertension, no diabetes, no use of medications that may affect body weight or energy intake
Flor-Alemany et al., 2020, Spain [57]	cross-sectional, 4	150	32.9 ± 4.6	1st trim	40% multiparous	24.9 ± 4.1	no	singleton pregnancy, not engaged in >300 min/wk of physical activity, 14–18 weeks at first evaluation, no pregnancy risk factors (e.g., vaginal bleeding)
Pauley et al., 2020, USA [46]	RCT (behavioral lifestyle intervention with calorie and physical activity goals to reduce GWG), 2	24	30.6 ± 3.2	1st trim	25% multiparous	31.8 ± 3.2	yes (overweight or obese)	18–40 yrs of age, with overweight or obesity, singleton pregnancy, >8 wks gestation, physician’s consent, English speaking, residing in or near Central Pennsylvania, no diabetes, no sever allergies or dietary restrictions, no contradictions to prenatal physical activity
Teixeira et al., 2020, Brazil [64]	cross-sectional, 4	245	median 25 (IQR 19–35)	any trim	58.2% previous pregnancy	median 24.6 (IQR 19.2–3.4)	no	attending prenatal clinic in the public health service, ≥18 years of age, not a shift worker, no use of illegal substances, no HIV or disease with toxoplasmosis, syphilis, varicella, rubella, or cytomegalovirus
Zhan et al., 2020, China [69]	cross-sectional, 4	7615	28.6 ± 4.3	1st trim	46.3% multiparous	22.0 ± 3.6 (pre-pregnancy)	no	part of larger cohort, ≥16 yrs of age, 5–13 wks gestation at recruitment, able to complete questionnaires, permanent residents in study locations, no serious chronic diseases, no psychosis
Betts et al., 2021, USA [70]	cohort, 3	373	30.8 ± 4.6	1st trim	not reported	26.6 ± 6.6	no	plan to deliver at specific hospital and remain in area until one year postpartum, singleton pregnancy, BMI > 18.5 kg/m^2^, 18–45 yrs of age, read and write English, access to internet and email, no psychiatric or eating disorder, no pre-existing diabetes or other medical condition contraindicating study participation
Du et al., 2021, China [54]	cross-sectional, 4	4352	median: 29 (IQR: 24–35)	1st and 2nd trim	not specified (gravidity-58% yes)	7.4% < 18.5, 69.7% 18.5–24.9, 18.5% 25–29.9, 4.4% > 30	no	18–45 yrs of age, <14 wks gestation at recruitment, resident of study area with no plans to move, intention to undergo prenatal care and delivery at specific hospital, no cognitive mental disorders, no pre-pregnancy diabetes or hypertension, no cardiovascular, liver, kidney, or autoimmune disease, singleton pregnancy
Gordon et al., 2021, USA [59]	cohort, 3	48	28.2 ± 4.9	3rd trim	64.6% multiparous	not reported	yes (history of major depression or bipolar disorder)	history of MDD or MPD but not meeting criteria for a current mood episode at enrollment, no primary Axis I diagnosis other than MDD or BPD, no diagnosed sleep disorder, no report of high-risk pregnancy, no current alcohol or drug dependence, no use of hypnotic medications, not a night shift worker
Quach et al., 2021, Vietnam [61]	cross-sectional, 4	400	median: 29 (IQR: 26–32)	any trim	63.5% multiparous	median: 20.5 (IQR: 19.2–22.4) (pre-pregnancy); 23.2 (IQR: 21.1–25.5 (at baseline)	no	≥18 yrs of age, no mental disorders preventing answering questions
Wang et al., 2021, USA [67]	cross-sectional, 4	108	median: 30 (IQR: 26–33)	2nd and 3rd trim	62% multigravida	55.6% normal weight, 25.0% overweight, 19.4% obese (pre-pregnancy)	no	>18 yrs of age, singleton pregnancy, <36 wks gestation at enrollment, pre-pregnancy body mass index of 18.5–40 kg/m^2^, planned to deliver at study hospital, comfortable reading and writing in English, able and willing to provide informed consent, no chronic medical conditions impacting body weight (e.g., pregestational diabetes, HIV/AIDS, chronic hepatitis, autoimmune disease), no current use of medication associated with. significant weight change or to treat opioid dependence, no previous weight loss surgery or current participation in a weight loss program

Melnyk levels of evidence: Level 2—experimental studies, Level 3—cohort studies, Level 4—cross-sectional studies; BMI: body mass index; trim: trimester; RCT: randomized controlled trial; GWG: gestational weight gain.

**Table 2:** Findings from studies with variables related to energy or nutrient intake (n = 12).

First Author, Year	Diet Var.	Measure/ Method	Variable Type/Specification	Time of Measurement (Weeks’ Gestation/Months Postpartum)	Sleep Var.	Measure(s) (Method)	Variable Type(s)/ Specification	Time of Measurement (Weeks’ Gestation/ Months Postpartum)	Statistical Analysis	Adjustment	Results
Diego et al., 2008 [52]	caffeine consumption	questionnaire (single item)	continuous: number of drinks per day	baseline (20–28 wks gestation)	Effectiveness; disturbance; supplementary sleep	Verran and Snyder-Halperin (1988) Sleep Scale	continuous	unclear	Pearson’s correlation	no	caffeine use was correlated with having less sleep effectiveness (r = −0.25, *p* < 0.01), with non-sig. findings for sleep disturbance and supplementary sleep
Chang et al., 2015 [50]	fat intake	24-item Rapid Food Screener (17 items for fat intake)	continuous (higher score indicates higher intake)	either 1st, 2nd, or 3rd trimester	duration; disturbance; quality; latency	Pittsburgh Sleep Quality Index (PSQI) (1 item for duration, 9 items for disturbance, 1 item for quality, 2 items for latency)	duration- categorical (>7 h, 6–7 h, 5–6 h, 3 < 5 h); disturbance- continuous (higher score indicates more sleep disturbance); quality- categorical (very good, good, bad, very bad); latency- continuous (higher score indicates longer sleep latency)	either 1st, 2nd, or 3rd trimester	Pearson’s correlation and path analysis	no	fat intake was correlated with nighttime sleep disturbance (*p* < 0.01), with non-sig. findings for sleep duration, quality, and latency; from path analysis, fat intake was associated with sleep disturbance in (standardized parameter estimate: 5.19, *p* < 0.01)
van Lee et al., 2017 [66]	energy intake; energy from discretionary foods	single 24 h recall face to face with 5 stage multiple pass method	continuous; sum of energy from caloric beverages (≥5 kcal; excluding plain water, diet soda, and unsweetened coffee, tea, and cow’s milk), local cakes, desserts, and snacks	at baseline (26–28 wks gestation)	quality; duration	Pittsburgh Sleep Quality Index (PSQI) (full index for quality, single item for duration)	quality—dichotomous (poor (defined as a global PSQI score > 5)/good); duration—dichotomous (short (defined as <6 h)/normal)	at baseline (26–28 wks gestation)	*t*-tests, chi-square tests, linear regression	yes ^1^	non-sig. findings
Wolynczyk-Gmaj et al., 2017 [68]	coffee drinking	structured non- validated questions	not specified	at baseline (35.0 + 3.7 wks gestation)	insomnia; sleepiness	Athens Insomnia Scale; Epworth Sleepiness Scale	insomnia—continuous (higher score indicating higher severity), dichotomous (yes/no (using 8 point cutoff)); sleepiness—continuous (higher score indicating greater sleepiness)	at baseline (35.0 ± 3.7 wks gestation)	*t*-tests, chi-square tests, logistic regression	yes ^2^	non-sig. findings
Phelan et al., 2018 [47]	behavioral lifestyle intervention with partial meal replacement	treatment group (randomly assigned)	dichotomous (meal replacement intervention/control)	at baseline and 35 wks gestation	duration	Actigraph (GT3X+) worn for 1 wk	continuous (h per day)	at baseline and 35 wks gestation	linear mixed effects models	yes ^3^	non-sig. findings
Bennett et al., 2019 [49]	energy intake, carbohydrate, protein, total fat, saturated fat, monounsaturated fat, polyunsaturated fat, fiber, and sugar intake; protein to carbohydrate ratio; glycemic index; glycemic load	Dietary Questionnaire for Epidemiological Studies version 2 (DQESv2), which is a food frequency questionnaire	energy intake—continuous (kJ), carbohydrates, protein, total fat, saturated fat, monounsaturated fat, polyunsaturated fat, fiber, sugars—continuous (% energy); protein to carbohydrate ratio (kJ:kJ); glycemic index- continuous; glycemic load—continuous	at baseline (1st, 2nd, or 3rd trimester)	sleeping behavior pattern	questionnaire (combined duration (on workdays and non-workdays) and sleep disorder symptoms (restless sleep past wk, difficulty falling asleep past month, severe tiredness past 12 months, difficulty sleeping past 12 months))	categorical: average sleep with no adverse sleep-related symptoms (LC1), average sleep with adverse sleep symptoms (LC2), short sleep with adverse sleep symptoms (LC3) (identified using latent class analysis)	at baseline (1st, 2nd, or 3rd trimester)	linear regression	yes ^4^	in crude models, compared to LC1, LC2 was associated with energy intake (B: 0.063, *p* < 0.05), monounsaturated fat intake (B: −0.034, *p* < 0.05), and glycemic load (B: 0.082, *p* < 0.01), while LC3 was associated with glycemic load only (B: 0.073, *p* < 0.05), non-sig. findings for all other dietary outcomes; after adjustment (including additional adjustment for pre-pregnancy BMI), LC2 was associated with fat intake (B: −0.032, *p* < 0.05), monounsaturated fat intake (B: −0.050, *p* < 0.01), and carbohydrate intake (B: 0.033, *p* < 0.05); non-sig. findings for all other dietary outcomes as well as for LC3 and all dietary outcomes
Loy et al., 2020 [60]	energy intake	24 h recall (5-stage multiple-pass interviewing technique)	continuous (kJ)	26–28 wks gestation	duration (nighttime); bedtime	questionnaire (single item); not specified	duration- dichotomous (short sleep (defined as < 6 h per night)/sufficient sleep); bedtime—continuous	26–28 wks gestation	*t*-tests	no	non-sig. findings
Du et al., 2020 [53]	caloric intake	24 h dietary recall (2 non-consecutive days)	continuous (kcal/day) and dichotomous (< or > 2300)	at baseline (<14 wks gestation)	duration	questionnaire (single (PSQI) item)	categorical: short (<7 h/night), normal (7–9 h/night), long (>9 h/night)	at baseline (<14 wks gestation)	chi-square (Kruskal–Wallis and Fisher’s exact)	no	non-sig. findings
Flanagan et al., 2021 [56]	energy intake	calculated as the sum of total daily energy expenditure and changes in energy stores by the energy-balance method	continuous (kcals across pregnancy)	13–16 and 35–37 wks gestation	change to habitual sleep (time spent in bed and total sleep time)	Actigraphy (GTX3+, wrist-worn for 6 consecutive nights) at two time points (change defined as change of one-half of the standard deviation of time spent in bed and total sleep time across 6 consecutive nights from early pregnancy)	categorical: increased, same, decreased	13–16 and 35–37 wks gestation	linear mixed models	yes ^5^	women who increased time in bed had lower energy intake across pregnancy compared to those who decreased (3078 ± 103 vs. 2538 ± 128, *p* < 0.01); non-sig. findings for total sleep time
Pauley et al., 2020 [46]	energy intake	estimated using a validated back-calculation method that uses weight (measured by participant with scale) and resting and active energy (physical activity—measured via activity monitor) each day	continuous (weekly average)	8–36 wks gestation	duration (nighttime and daytime); awakenings	wrist worn activity monitor (Jawbone)	duration—continuous (weekly average min per day); awakenings—continuous (weekly average number per night)	8–36 wks gestation	multi-level modeling	yes ^6^	non-sig. findings
Du et al., 2021 [54]	caloric intake	24 h dietary recall (2 non-consecutive days)	dichotomous (< or > 2300)	at baseline (<14 wks gestation)	quality	Pittsburgh Sleep Quality Index (PSQI)	dichotomous: poor (defined as >5 on the PSQI)/not	at baseline (<14 wks gestation)	chi-square tests and logistic regression	yes ^7^	non-sig. findings
Wang et al., 2021 [67]	sugar-sweetened beverage consumption	24 h recall (3 days (2 weekdays and 1 weekend day), via phone, interview with multiple pass technique)	continuous: average 8 oz servings per day	baseline (median: 23.9 wks (IQR: 18.9–30.6))	duration; quality	Pittsburgh Sleep Quality Index (PSQI)	duration—dichotomous (short sleep (defined as <7 h per night)/sufficient sleep); quality—dichotomous (poor (defined as PSQI score >5)/not)	baseline (median: 23.9 wks (IQR: 18.9–30.6))	logistic regression	yes ^8^	each additional serving of a sugar-sweetened beverage was associated with higher odds of short sleep (adjusted OR:1.6, 1.1–2.5) and poor sleep quality (adjusted OR:2.1, 1.2–3.6)

^1^: alcohol intake during pregnancy, physical activity during pregnancy, household income, education level, ethnicity, energy intake, age, parity; ^2^: depressive symptoms, legs tingling, snoring, age, hyperarousal, nightmares, myoclonus; ^3^: gestational age at enrollment, age, income, ethnicity, parity, BMI category, site); ^4^: area of residence, BMI, depression, difficulty managing on income, education level, parity; ^5^: baseline BMI; ^6^: study group; ^7^: stillbirth history, induced abortion history, health-related quality of life, physical activity, smoking, folic acid supplementation for 3 months before pregnancy; ^8^: marital status, education, and financial strain.

**Table 3:** Findings from studies with variables related to dietary patterns (n = 9).

First Author, Year	Diet Var.	Measure/ Method	Variable Type/ Specification	Time of Measurement (Weeks’ Gestation/ Months Postpartum)	Sleep Var.	Measure(s) (Method)	Variable Type(s)/Specification	Time of Measurement (Weeks’ Gestation/ Months Postpartum)	Statistical Analysis	Adjustment	Results
Chang et al., 2015 [50]	fruit and vegetable intake	24-item Rapid Food Screener (7 items for fruit and vegetable intake)	continuous (higher score indicates higher intake)	either 1st, 2nd, or 3rd trimester	duration; disturbance; quality; latency	Pittsburgh Sleep Quality Index (PSQI) (1 item for duration, 9 items for disturbance, 1 item for quality, 2 items for latency)	duration- categorical (>7 h, 6–7 h, 5–6 h, 3 < 5 h); disturbance- continuous (higher score indicates more sleep disturbance); quality—categorical (very good, good, bad, very bad); latency—continuous (higher score indicates longer sleep latency)	either 1st, 2nd, or 3rd trimester	Pearson’s correlation and path analysis	no	non-sig. correlations between fruit and vegetable intake with sleep duration, quality, latency, and disturbance; from path analysis, fruit and vegetable intake was associated with sleep latency (standardized parameter estimate: 1.47, *p* < 0.05)
Duke et al., 2017 [55]	fruit and vegetable consumption	questionnaire (4 items: consumption of fruit, dark green vegetables, orange-colored vegetables, and other vegetables during the past month)	continuous (consumption per day for each item and summed for total daily fruit and vegetable consumption)	not specified	duration	questionnaire (single item)	continuous (h per day) and categorical (inadequate sleep—<6 h, recommended sleep—7–9 h, over recommended sleep—>10 h)	not specified	analysis of variance, linear and logistic regression	yes ^1^	in crude analyses, sleep recommendation categories were associated with other vegetable (*p* < 0.01), fruit (*p* < 0.01), and total fruit and vegetable (*p* < 0.05) consumption; in adjusted models, orange (B: −0.19 (−0.38, −0.01)) and green (B: −0.20 (−0.33, −0.08)) vegetable consumption were associated with sleep duration, non-sig. findings for total and other daily fruit and vegetable consumption; odds of meeting or exceeding sleep time recommendations increased slightly with each unit increase in total fruit and vegetable consumption (OR:1.05 (1.002,1.092) and for every unit increase in fruit consumption (OR:1.12 (1.038,1.208), non-sig. findings for green, orange, and other vegetable consumption
van Lee et al., 2017 [66]	diet quality; dietary pattern	single 24 h recall face to face with 5 stage multiple pass method	quality—continuous (using the Healthy Eating Index for pregnant women in Singapore (HEI-SGP)) and dichotomous (poor/good); pattern—categorical (vegetables-fruit-rice, seafood-noodles, pasta-cheese-meat);	at baseline (26–28 wks gestation)	quality; duration	Pittsburgh Sleep Quality Index (PSQI) (full index for quality, single item for duration)	quality—dichotomous (poor (defined as a global PSQI score >5)/good); duration—dichotomous (short (defined as <6 h)/normal)	at baseline (26–28 wks gestation)	*t*-tests, chi-square tests, linear regression	yes ^2^	In adjusted analyses, good sleep quality compared to poor sleep quality was associated with better diet quality (mean HEI-SGP 54.6 vs. 52.0, *p* < 0.05), greater adherence to the vegetables-fruit-rice pattern (mean 0.03 vs. −0.15, *p* < 0.05), and lesser adherence to the seafood-noodle pattern (mean −0.14 vs. 0.03, *p* < 0.05, also significant after further adjustment for anxiety); non-sig. findings for pasta-cheese-meat pattern; non-sig. findings for short compared to normal sleep for diet quality, nor any dietary pattern
Gontijo et al., 209 [58]	quality (overall and individual components)	3 24 h recalls on nonconsecutive days including one on the weekend (by interview)	continuous (average consumption across 3 days, evaluated using the Brazilian Health Eating Index-Revised (BHEI-R), overall and 12 components)	at baseline (<12 wks gestation)	chronotype	questionnaire (items asking about usual bedtime, usual wake time, sleep-onset latency, and usual sleep duration on weekdays and weekends during pregnancy)	categorical—morning type, intermediate type, evening type (using mid-sleep time on free days on weekends, with a further correction for sleep debt (calculated as the difference between average sleep duration on weekend and weekdays))	at baseline (<12 wks gestation)	linear regression	yes ^3^	chronotype was associated with total grains (B: 0.169, *p* < 0.01) and total fruit (B: −0.236, *p* < 0.05), non-sig. findings for all other Brazilian Healthy Eating Index Revised components
Du et al., 2021 [53]	type of diet	not specified	categorical (balanced, more meat, veggie-rich, vegan)	at baseline (<14 wks gestation)	duration	questionnaire (single (PSQI) item)	categorical: short (<7 h/night), normal (7–9 h/night), long (>9 h/night)	at baseline (<14 wks gestation)	chi-square (Kruskal–Wallis and Fisher’s exact)	no	type of diet was associated with sleep duration (*p* < 0.05), with short and long sleep durations more likely to be observed in vegans
Flanagan et al., 2021 [56]	diet quality	2015 Healthy Eating Index (HEI) based on 6 days of food and plate waste photographs (SmartIntake phone App)	continuous	13–16 and 35–37 wks gestation	change to habitual sleep (time spent in bed and total sleep time)	Actigraphy (GTX3+, wrist-worn for 6 consecutive nights) at two time points (change defined as change of one-half of the standard deviation of time spent in bed and total sleep time across 6 consecutive nights from early pregnancy)	categorical: increased, same, decreased	13–16 and 35–37 wks gestation	linear mixed models	yes ^4^	non-sig. findings
Flor-Alemany et al., 2020 [57]	Mediterranean diet adherence and components	Food Frequency Questionnaire (by interview)	Mediterranean adherence score- continuous and tertile (using Mediterranean Food Pattern, total score and 8 elements: olive oil, fiber, fruit, vegetables, fish, cereals, meat, alcohol)	16 and 34 wks gestation	sleep quality	Pittsburgh Sleep Quality Index (PSQI)	continuous (lower values indicate better sleep quality) and dichotomous (good/bad using cutoff of 5)	16 and 34 wks gestation	Spearman correlation, Kruskal–Wallis test	no	Mediterranean Food Pattern as a continuous variable was associated with better sleep quality at 16 (*p* < 0.05) and 34 wks (*p* < 0.01) gestation; similar findings for tertiles (*p* < 0.05 at 16 wks and *p* < 0.01 at 34 wks); fruit consumption was associated with better sleep quality at 16 wks gestation (*p* < 0.01); olive oil consumption and Mediterranean Diet adherence were associated with better sleep quality at 16 (*p* < 0.05) and 34 wks (*p* < 0.05) gestation; red meat and subproduct consumption was associated with worse sleep quality at 34 wks gestation (*p* < 0.05)
Zhan et al., 2020 [69]	dietary pattern (plant-based, vitamin-rich, high-fat, animal protein-rich, bean products)	Qualitative Food Frequency Questionnaire (Q-FFQ), with patterns derived via factor analysis	quartiles	at baseline (5–13 wks gestation)	quality	Pittsburgh Sleep Quality Index (PSQI)	dichotomous: sleep disturbance (defined as >5 on the PSQI)/normal	at baseline (5–13 wks gestation)	logistic regression	yes ^5^	in the fully adjusted model, participants with the highest quartile in plant-based (OR: 0.80, 0.68–0.93) and vitamin-rich (OR: 0.76, 0.65–0.89) patterns had less sleep disturbance, while those in the highest quartile for high-fat pattern (OR:1.43, 1.22–1.67) has more sleep disturbance; non-sig. findings for animal protein-rich and bean products patterns in the fully adjusted model, but bean product patterns was associated with less sleep disturbance in the crude and partially adjusted models
Du et al., 2021 [54]	type of diet	not specified	categorical (balanced, more meat, more vegetables, vegetarian)	at baseline (<14 wks gestation)	quality	Pittsburgh Sleep Quality Index (PSQI)	dichotomous: poor (defined as >5 on the PSQI)/not	at baseline (<14 wks gestation)	chi-square tests and logistic regression	yes ^6^	those with a vegetarian diet type (OR:2.18, 1.54–3.08) and those with a more vegetables diet type (OR:1.32, 1.14–1.52) were more likely to have poor sleep compared to those with a balanced diet

^1^: age, race/ethnicity, education, physical activity, marital status, income, and employment; ^2^: alcohol intake during pregnancy, physical activity during pregnancy, household income, education level, ethnicity, energy intake, age, parity; ^3^: age, BMI, maternal schooling, frequency of nausea in the last 30 days; ^4^: baseline BMI; ^5^: age, pre-pregnancy BMI, parity, SES, region, tobacco exposure, alcohol consumption, physical activity; ^6^: stillbirth history, induced abortion history, health-related quality of life, physical activity, smoking, folic acid supplementation for 3 months before pregnancy.

**Table 4:** Findings from studies with variables related to eating behaviors (n = 10).

First Author, Year	Diet Var.	Measure/Method	Variable Type/ Specification	Time of Measurement (Weeks’ Gestation/ Months Postpartum)	Sleep Var.	Measure(s) (Method)	Variable Type(s)/ Specification	Time of Measurement (Weeks’ Gestation/ Months Postpartum)	Statistical Analysis	Adjustment	Results
Allison et al., 2012 [48]	nighttime eating; disordered eating; cognitive restraint over eating; overeating at meals; snacking between meals; snacking after dinner; eating due to physical hunger; eating due to cravings; eating when anxious, bored, stressed, angry, depressed/upset, or alone	Night Eating Questionnaire (NEQ); Eating Disorders Examination-Questionnaire (EDE-Q) sections on objective overeating, binge eating, and purging; item 51 of the Eating Inventory to assess cognitive restraint over eating; Weight and Lifestyle Inventory	continuous: higher scores indicating more severe pathology	baseline (14–24 wks gestation)	duration; latency; quality	Pittsburgh Sleep Quality Index (PSQI) questions 1–6	duration—continuous: h/night; latency—continuous: minutes; quality—categorical (very bad, fairly bad, fairly good, very good)	baseline (14–24 wks gestation)	Pearson’s correlation	no	sleep duration was correlated with night eating (r = −0.28, *p* < 0.01), with non-sig. findings for restraint, overeating episodes, and binge episodes; latency was correlated with night eating (r = 0.25, p < 0.05) and overeating episodes (r = 0.24, *p* < 0.05), with non-sig. findings for restraint and binge episodes; quality was correlated with night eating (r = −0.43, *p* < 0.01), overeating episodes (r = −0.25, *p* < 0.05), and binge episodes (r = −0.26, *p* < 0.01), with non-sig. findings for restraint
Ulman et al., 2012 [65]	binge eating symptoms	questionnaire (items addressing DSM-IV eating disorder criteria, questions addressing both eating an unusually large amount of food and feeling out of control)	categorical: binge eating disorder symptoms before and during pregnancy, symptoms before pregnancy that remitted during pregnancy, incident binge eating disorder symptoms during pregnancy, no reported symptoms before or during pregnancy	at baseline (~17.1 wks gestation)	sleep problems during first 18wks of pregnancy; sleep duration during the 3rd trimester; sleep disatisfaction 18 months after birth	questionnaire (single items)	sleeping problems- dichotomous (yes/no); duration- categorical (<6 h, 6–9 h, >10 h); sleep disatisfaction- yes/no	sleeping problems—median 17.1 wks gestation); duration—median 30.1 wks gestation; satisfaction—median 18.7 months postpartum	logistic regression	yes ^1^	in both crude and adjusted models, all binge eating disorder symptom groups were more likely to report sleep problems during the first 18wks of pregnancy than the no symptoms group (adjusted ORs:1.26–1.42, *p* < 0.05); in crude models, all binge eating disorder symptom groups were more likely to sleep 10+ h or <6 h than the no symptoms groups, with the association persisting in the adjusted model for the incident binge eating disorder symptoms group (adjusted OR for 10+ h: 1.49, *p* < 0.01; adjusted OR for <6 h:1.58, *p* < 0.01); in both crude and adjusted models, all binge eating disorder symptom groups had higher odds of reporting sleep dissatisfaction at 18 months after birth than the no symptoms group (adjusted ORs:1.28–1.47, *p* < 0.01)
van Lee et al., 2017 [66]	longest night-time fasting interval; frequency of consumption occasions; nighttime eating	single 24 h recall face to face with 5 stage multiple pass method	continuous: longest fasting interval from 19:00 h to 06:59 h; number of eating occasion providing ≥50 kcal with 15 min time interval between occasions	at baseline (26–28 wks gestation)	quality; duration	Pittsburgh Sleep Quality Index (PSQI) (full index for quality, single item for duration)	quality- dichotomous (poor (defined as a global PSQI score >5)/good); duration—dichotomous (short (defined as <6 h)/normal)	at baseline (26–28 wks gestation)	*t*-tests, chi-square tests, linear regression	yes ^2^	non-sig. findings
Wolynczyk-Gmaj et al., 2017 [68]	eating at night	structured non-validated questions	not specified	at baseline (35.0 + 3.7 wks gestation)	insomnia; sleepiness	Athens Insomnia Scale; Epworth Sleepiness Scale	insomnia- continuous (higher score indicating higher severity), dichotomous (yes/no (using 8 point cutoff)); sleepiness—continuous (higher score indicating greater sleepiness)	at baseline (35.0 ± 3.7 wks gestation)	*t*-tests, chi-square tests, logistic regression	yes ^3^	women with insomnia during pregnancy were more likely to eat at night (chi-square = 18.15, df = 1, *p* < 0.01); from logistic regression, after adjustment, eating at night was associated with insomnia during pregnancy (OR: 2.935, 1.22, 7.07)
Gontijo et al., 2019 [58]	nightly fasting; eating duration; time of the first meal; time of the last meal; number of meals	3 24 h recalls on nonconsecutive days including one on the weekend (by interview)	continuous	at baseline (<12 wks gestation)	chronotype	questionnaire (items asking about usual bedtime, usual wake time, sleep-onset latency, and usual sleep duration on weekdays and weekends during pregnancy)	categorical—morning type, intermediate type, evening type (using mid-sleep time on free days on weekends, with a further correction for sleep debt (calculated as the difference between average sleep duration on weekend and weekdays))	at baseline (<12 wks gestation)	linear regression	yes ^4^	non-sig. findings
Loy et al., 2020 [60]	nighttime eating; number of eating episodes	24 h recall (5-stage multiple-pass interviewing technique)	nighttime eating—dichotomous: yes/no (defined as consuming >50% of total energy intake from 7 a.m.–7 p.m.); eating episodes—continuous (defined as events that provide >120 kJ (~50 kcal) with time intervals between eating episodes of >15 min)	26–28 wks gestation	duration (nighttime); bedtime	questionnaire (single item); not specified	duration—dichotomous (short sleep (defined as <6 h per night)/sufficient sleep); bedtime—continuous	26–28 wks gestation	*t*-tests	no	those with night-eating had later bedtimes (2307 ± 0133 vs. 2336 ± 0222, *p* < 0.05); non-sig. finding for sleep duration and daily eating episodes
Flanagan et al., 2021 [56]	disinhibition, dietary restraint, perceived hunger, food cravings, mindful eating	Eating Inventory (EI, for disinhibition, dietary restraint, and perceived hunger); Food Craving Inventory (FCI); Mindful Eating Questionnaire (MEQ)	continuous	13–16 and 35–37 wks gestation	change to habitual sleep (time spent in bed and total sleep time)	Actigraphy (GTX3+, wrist-worn for 6 consecutive nights) at two time points (change defined as change of one-half of the standard deviation of time spent in bed and total sleep time across 6 consecutive nights from early pregnancy)	categorical: increased, same, decreased	13–16 and 35–37 wks gestation	linear mixed models	yes ^5^	women who increased time in bed reported an increase in food craving (23.8 ± 4.4% increase) compared to those who decreased (5.07 ± 6.4% increase, *p* = 0.05) across pregnancy; non-sig. findings for mindful eating, disinhibition, dietary restraint, and perceived hunger; non-sig. findings for total sleep time
Teixeira et al., 2020 [64]	food cravings	Food Craving Questionnaire Trait (FCQ-T) total score and 9 subscales (intentions and plans to consume food, anticipation of positive reinforcement that may result from eating, anticipation of relief from negative states and feeling as a result of eating, lack of control over eating, thoughts and preoccupations with food, craving as a physiological state, emotions that may be experienced before or during food craving, guilt from craving and/or from giving into them; Food Craving Questionnaire States (FCQ-S) total score and 5 subscales (intense desire to eat, anticipation of positive reinforcement that may result from eating, anticipation of relief from negative states and feelings as a result of eating, lack of control of eating, craving as a physiological state)	continuous (higher scores indicate more frequent and intense cravings)	4–40 wks	chronotype	questionnaire (reported usual bed and wake times on weekdays and weekends)	continuous and categorical- morning type, intermediate type, non-evening type (morning and intermediate), evening type (calculated using mid-sleep time on free days with a further correction for calculated sleep debt)	4–40 wks	generalized linear models	yes ^6^	evening types had higher scores on 2 of 9 FCQ-T subscales: anticipation of relief from negative states and feelings as a result of eating compared to morning (B: 0.180, *p* < 0.5) and non-evening types (B: 0.150, *p* < 0.05) and anticipation of positive reinforcement that may result from eating compared to morning types (B: 0.132, *p* < 0.05); evening types had lower scores on 2 of 5 FCQ-S subscales: intense desire to eat compared to morning (B: −0.188, *p* < 0.05) and non-evening types (B: −0.184, *p* < 0.01) and anticipation of positive reinforcement that may result from eating than non-evening types (B: −0.152, *p* < 0.05); chronotype score was associated with anticipation of relief from negative states and feelings as a result of eating (*p* < 0.01), anticipation of positive reinforcement that may result from eating as a usual behavior (*p* < 0.05), and intense desire to eat as a sporadic behavior (*p* < 0.05)
Betts et al., 2021 [70]	addictive-like eating; hedonic hunger; cravings (frequency and intensity)	Yale Food Addiction Scale; Power of Food Scale; items developed by study investigators (assessed most craved food foods and frequency and intensity of overall and specific food cravings)	addictive-like eating—categorical (for cross-sectional analyses): did not meet threshold for any items, met threshold for 1 item, met threshold for 2 or more items, continuous (for change scores); hedonic hunger—continuous (calculated as the mean of three component scores); cravings—frequency and intensity calculated as highest response across foods	baseline (<12 wks gestation) and 6 months postpartum (cravings only assessed during pregnancy)	quality	Pittsburgh Sleep Quality Index (PSQI)	continuous	baseline (<12 wks gestation) and 6 months postpartum	Pearson’s correlation, linear and logistic regression	yes ^7^	during pregnancy sleep quality was correlated with hedonic hunger (r = 0.15, *p* < 0.05), addictive-like eating (r = 0.20, *p* < 0.05), craving frequency (r = 0.23, *p* < 0.01) and craving strength (r = 0.24, *p* < 0.01); non-sig. findings for postpartum correlations; in regression analyses, worse sleep quality during pregnancy was associated with greater addictive-like eating (OR:1.09, 1.00–1.18), hedonic hunger (B: 0.03 (SE: 0.01)), and more frequent (B: 0.11 (SE: 0.03)) and intense (B: 0.13 (SE: 0.03)) cravings during pregnancy, while worse sleep quality postpartum was associated with greater addictive-like eating only (OR:1.13, 1.03–1.23); non-sig. findings for association between change in sleep quality with change in addictive-like eating or hedonic hunger
Quach et al., 2021 [61]	meal-to-bed time (daytime, nighttime, daytime and/or nighttime)	questionnaire (single item)	dichotomous: short (sleep within 2 h of finishing a meal on more than 2/3 of days during wk)/not short	baseline (median: 22.1 (IQR: 12.0–33.0))	insomnia (reflux-related)	questionnaire (single item)	dichotomous: yes/no (during past 7 days)	baseline (median: 22.1 (IQR: 12.0–33.0))	logistic regression	yes ^8^	in both univariate (OR:4.60, 1.64–12.92) and multivariate analyses (OR:3.68, 1.14–11.85), meal-to-bed time was a risk factor for reflux-related insomnia

^1^: gestation age of at birth, sex of child, parity, combined parental income, smoking status during pregnancy, mother’s education, maternal anxiety and depression during pregnancy, maternal age, maternal BMI; ^2^: alcohol intake during pregnancy, physical activity during pregnancy, household income, education level, ethnicity, energy intake, age, parity; ^3^: depressive symptoms, legs tingling, snoring, age, hyperarousal, nightmares, myoclonus; ^4^: age, BMI, maternal schooling, frequency of nausea in the last 30 days; ^5^: baseline BMI; ^6^: age, trimester, sleep quality, sleep time, nausea; ^7^: age, marital status, education, income-to-poverty ratio; ^8^: habit of drinking alcohol, previous history with typical reflux symptoms.

**Table 5:** Findings from studies with fetal/infant outcomes (n = 5).

First Author, Year	Diet Var.	Measure/ Method	Variable Type/ Specification	Time of Measurement (Weeks’ Gestation/ Months Postpartum)	Sleep Var.	Measure(s) (Method)	Variable Type(s)/ Specification	Time of Measurement (Weeks’ Gestation/ Months Postpartum)	Statistical Analysis	Adjustment	Results
Devoe et al., 1993 [51]	maternal caffeine consumption	questionnaire (multiple items)	dichotomous: high consumer (>500 mg/day) and low consumers (<200 mg/day)	baseline	infant fetal behavioral state	2 h ultrasonographic observations of body, breathing, and eye movements	categorical: quiet sleep, active sleep, quiet wakefulness, arousal, no state	biweekly from 30–40 wks gestation	analysis of variance	no	infants in both groups spent similar mean time in a quiet sleep state, but infants of high consumers spend less mean time in active sleep
Diego et al., 2008 [52]	maternal caffeine consumption	questionnaire (single item)	continuous: number of drinks per day	baseline (20–28 wks gestation)	infant sleep-wake behavior; maternal sleep effectiveness and disturbance	45 min live recording during inter-feeding interval; Verran and Snyder-Halperin (1988) Sleep Scale	categorical using Thoman’s Sleep State Criteria to assign state: quiet sleep, active sleep, REM, drowsy, awake alert, fussy, crying; continuous	within 24 h post-delivery; unclear	Pearson’s correlation	no	newborns of women who used more caffeine spent more time REM sleep (r = 0.24, *p* < 0.01, with non-sig. findings for quiet sleep, active sleep, drowsy, and awake alert states; caffeine use was correlated with having less sleep effectiveness (r = −0.25, *p* < 0.01), with non-sig. findings for sleep disturbance and supplementary sleep
Santos et al., 2012 [62]	maternal caffeine consumption	questionnaire (asked about instant and ground coffee and mate (a hot tea like beverage))	dichotomous: heavy consumption (>300 mg/day) or not	at hospital after delivery	infant daytime sleep duration; night wakings	maternal report	daytime sleep duration- categorical (tertiles based on hours/day); night wakings—dichotomous (yes/no for >3 times per night in the previous 15 days)	3 months of age	Poisson regression	yes ^1^	non-sig. findings
Sugimori et al., 2019 [63]	maternal fermented food intake	Food Frequency Questionnaire (items about miso soup, yogurt, cheese, and Japanese fermented soybean intake)	quartiles based on estimated daily intake	2nd and 3rd trimesters	infant duration	maternal report	dichotomous: <11 h or >11 h	1 year of age	logistic regression	yes ^2^	in crude and adjusted models, miso intake was associated with risk of sleeping <11 h for quartiles 2, 3, and 4, compared to 1 (adjusted ORs: 0.87–0.92, *p* < 0.01); yogurt intake was only associated with risk of sleeping <11 h in the crude analysis for quartile 3 compared to 1 (OR:0.91 (0.85, 0.98); cheese intake was associated with risk of sleeping <11 h in the crude analysis for quartiles 2 (OR:0.92 (0.86, 0.99) and 3 (OR:0.88 (.82, 0.95) compared to 1, but just for quartile 3 in the adjusted model (OR: 0.92 (0.85, 0.99); fermented bean intake was only associated with risk of sleeping <11 h in the crude analysis for quartile 3 compared to 1 (OR: 0.91 (0.85, 0.98)
Gordon et al., 2021 [59]	infant feeding status	daily diaries	categorical: exclusive breastfeeding, mixed (breastfeeding and formula), no breastfeeding, and dichotomous: any breastfeeding (exclusive or mixed)/no breastfeeding	2 and 16 wks postpartum	maternal sleep disturbance/efficiency; chronotype	disturbance/efficiency- actigraphy (Micro Motionlogger Watch (AMI), wrist worn for one week) complimented by sleep diary) and Pittsburgh Sleep Quality Index (PSQI); chronotype-Horne-Ostberg Morningness-Eveningness Questionnaire (MEQ)	objective disturbance-efficiency- dichotomous: lower sleep efficiency/higher sleep efficiency (based on median split); subjective disturbance/efficiency- continuous; chronotype-continuous	33 wks gestation	chi-square, *t*-tests	no	mothers with low sleep efficiency during pregnancy were less likely than high sleep efficiency mothers to initiate breastfeeding (45.8% vs. 16.7% no breastfeeding, *p* < 0.05); trend for similar results at 16 wks postpartum (chi-square (df = 2) = 4.61, *p* = 0.10); non-sig. findings for subjective sleep disturbance and chronotype with feeding status

^1^: maternal age, skin color, schooling, parity, smoking, alcohol consumption, child’s gender, family income; ^2^: energy intake, maternal age, previous deliveries, BMI at 1 month after delivery, maternal education, annual household income, marital status at 6 months after delivery, alcohol intake at 1 month after delivery, smoking status at 1 month after delivery, employment status at 1 yr after delivery, infant sex, daycare attendance, infant sleep location, birth weight, gestational age, presence of any disease, month of birth, yogurt intake at 1 yr old, cheese intake at 1 yr old.

## Appendix A. Search Strategy

PubMed:

1	Birthing Centers/(819)
2	Maternal Health/(1765)
3	exp Maternal Health Services/(52223)
4	Maternal-Fetal Relations/(925)
5	Maternal-Child Nursing/(1939)
6	exp Pregnancy/(920493)
7	exp Pregnancy Complications/(438930)
8	Pregnant Women/or Surrogate Mothers/(11325)
9	exp delivery, obstetric/(83094)
10	(antenatal or gestat* or maternal* or maternity or perinatal or pregnan* or prenatal or surroga*).ti,ab,kf. (933301)
11	or/1–10 (1331767)
12	exp Sleep/(83550)
13	exp Sleep Wake Disorders/(94508)
14	Sleep Medicine Specialty/(387)
15	sleepiness/(455)
16	(sleep* or slept or somnolen*).ti,ab,kf. (201971)
17	(dream* or REM or rapid eye movement* or non-REM or NREM).ti,ab,kf. (31925)
18	(drowsy or drowsiness).ti,ab,kf. (7064)
19	or/12–18 (248547)
20	exp “diet, food, and nutrition”/(1778723)
21	exp Plants, Edible/(46593)
22	exp Dietary Services/(7054)
23	exp “Feeding and Eating Disorders”/(31946)
24	exp Nutrition Therapy/(104976)
25	nutritional sciences/or dietetics/or nutrigenomics/(18726)
26	exp Nutrition Policy/(11681)
27	exp Food Industry/(190037)
28	(diet* or supplement*).ti,ab,kf. (875322)
29	(eat or eaten or eats or eating or ate or cook*).ti,ab,kf. (143528)
30	(food* or fed or feed*).ti,ab,kf. (1035612)
31	(calorie* or caloric or high-calorie* or low-calorie* or high-caloric* or low-caloric*).ti,ab,kf. (46685)
32	(macro-nutrient* or macronutrient* or nutrition* or nutrient* or nutritive*).ti,ab,kf. (449174)
33	(consume? or consuming or consumption).ti,ab,kf. (475613)
34	energy.ti,ab,kf. (687974)
35	(intake or ingest*).ti,ab,kf. (373987)
36	(carbohydrate* or high-carb* or low-carb* or sugar*).ti,ab,kf. (266900)
37	(fat or fats or fat-rich or fat-heavy or fatty or high-fat* or low-fat* or omega-3* or omega-6* or oil or oils).ti,ab,kf. (657162)
38	(amino acid* or high-protein* or low-protein* or protein*).ti,ab,kf. (3425839)
39	(cholesterol or high-cholesterol or HDL or low-cholesterol or LDL or lipoprotein*).ti,ab,kf. (334576)
40	(dairy or fish or fruit* or vegetable* or meat or seafood or vegan* or vegetarian* or nonvegetarian* or non-vegetarian* or paleo* or pescatarian or keto* or mediterranean or meal or meals).ti,ab,kf. (648313)
41	(butter* or ghee* or milk*).ti,ab,kf. (149412)
42	(superfood* or probiotic*).ti,ab,kf. (28331)
43	(hunger or hungry or malnutrition).ti,ab,kf. (55056)
44	or/20–43 (7595460)
45	11 and 19 and 44 (2520)

EMBASE:

(‘maternity ward’/exp OR ‘maternal care’/exp OR ‘maternal health service’/exp OR ‘mother fetus relationship’/exp OR ‘maternal child health care’/exp OR ‘pregnancy’/exp OR ‘pregnancy disorder’/exp OR ‘pregnant woman’/exp OR ‘surrogate mother’/exp OR ‘obstetric delivery’/exp OR antenatal:ti,ab,kw OR gestat*:ti,ab,kw OR maternal*:ti,ab,kw OR maternity:ti,ab,kw OR perinatal:ti,ab,kw OR pregnan*:ti,ab,kw OR prenatal:ti,ab,kw OR surroga*:ti,ab,kw) AND (‘sleep’/exp OR ‘sleep disorder’/exp OR ‘sleep medicine’/exp OR ‘somnolence’/exp OR sleep*:ti,ab,kw OR slept:ti,ab,kw OR somnolen*:ti,ab,kw OR dream*:ti,ab,kw OR rem:ti,ab,kw OR ‘rapid eye movement*’:ti,ab,kw OR ‘non-rem’:ti,ab,kw OR nrem:ti,ab,kw OR drowsy:ti,ab,kw OR drowsiness:ti,ab,kw) AND (‘nutrition’/exp OR ‘edible plant’/exp OR ‘digestive function’/exp OR ‘dietary service’/exp OR ‘nutrition service’/exp OR ‘eating disorder’/exp OR ‘diet therapy’/exp OR ‘nutritional science’/exp OR ‘dietetics’/exp OR ‘nutrigenomics’/exp OR ‘nutrition policy’/exp OR ‘food handling’/exp OR ‘food insecurity’/exp OR diet*:ti,ab,kw OR supplement*:ti,ab,kw OR eat:ti,ab,kw OR eaten:ti,ab,kw OR eats:ti,ab,kw OR eating:ti,ab,kw OR ate:ti,ab,kw OR cook*:ti,ab,kw OR food*:ti,ab,kw OR fed:ti,ab,kw OR feed*:ti,ab,kw OR calorie*:ti,ab,kw OR caloric:ti,ab,kw OR ‘high-calorie*’:ti,ab,kw OR ‘low-calorie*’:ti,ab,kw OR ‘high-caloric*’:ti,ab,kw OR ‘low-caloric*’:ti,ab,kw OR ‘macro-nutrient*’:ti,ab,kw OR macronutrient*:ti,ab,kw OR nutrition*:ti,ab,kw OR nutrient*:ti,ab,kw OR nutritive*:ti,ab,kw OR consume$:ti,ab,kw OR consuming:ti,ab,kw OR consumption:ti,ab,kw OR energy:ti,ab,kw OR intake:ti,ab,kw OR ingest*:ti,ab,kw OR carbohydrate*:ti,ab,kw OR ‘high-carb*’:ti,ab,kw OR ‘low-carb*’:ti,ab,kw OR sugar*:ti,ab,kw OR fat:ti,ab,kw OR fats:ti,ab,kw OR ‘fat-rich’:ti,ab,kw OR ‘fat-heavy’:ti,ab,kw OR fatty:ti,ab,kw OR ‘high-fat*’:ti,ab,kw OR ‘low-fat*’:ti,ab,kw OR ‘omega-3*’:ti,ab,kw OR ‘omega-6*’:ti,ab,kw OR oil:ti,ab,kw OR oils:ti,ab,kw OR ‘amino acid*’:ti,ab,kw OR ‘high-protein*’:ti,ab,kw OR ‘low-protein*’:ti,ab,kw OR protein*:ti,ab,kw OR cholesterol:ti,ab,kw OR ‘high-cholesterol’:ti,ab,kw OR hdl:ti,ab,kw OR ‘low-cholesterol’:ti,ab,kw OR ldl:ti,ab,kw OR lipoprotein*:ti,ab,kw OR dairy:ti,ab,kw OR fish:ti,ab,kw OR fruit*:ti,ab,kw OR vegetable*:ti,ab,kw OR meat:ti,ab,kw OR seafood:ti,ab,kw OR vegan*:ti,ab,kw OR vegetarian*:ti,ab,kw OR nonvegetarian*:ti,ab,kw OR ‘non-vegetarian*’:ti,ab,kw OR paleo*:ti,ab,kw OR pescatarian:ti,ab,kw OR keto*:ti,ab,kw OR mediterranean:ti,ab,kw OR meal:ti,ab,kw OR meals:ti,ab,kw OR butter*:ti,ab,kw OR ghee*:ti,ab,kw OR milk*:ti,ab,kw OR superfood*:ti,ab,kw OR probiotic*:ti,ab,kw OR hunger:ti,ab,kw OR hungry:ti,ab,kw OR malnutrition:ti,ab,kw) NOT ( [animals]/lim NOT [humans]/lim) AND [english]/lim

CINAHL:

S48	S11 AND S18 AND S47
S47	S19 OR S20 OR S21 OR S22 OR S23 OR S24 OR S25 OR S26 OR S27 OR S28 OR S29 OR S30 OR S31 OR S32 OR S33 OR S34 OR S35 OR S36 OR S37 OR S38 OR S39 OR S40 OR S41 OR S42 OR S43 OR S44 OR S45 OR S46
S46	TI (hunger or hungry or malnutrition) OR AB (hunger or hungry or malnutrition)
S45	TI (superfood* or probiotic*) OR AB (superfood* or probiotic*)
S44	TI (butter* or ghee* or milk*) OR AB (butter* or ghee* or milk*)
S43	TI (dairy or fish or fruit* or vegetable* or meat or seafood or vegan* or vegetarian* or nonvegetarian* or non-vegetarian* or paleo* or pescatarian or keto* or mediterranean or meal or meals) OR AB (dairy or fish or fruit* or vegetable* or meat or seafood or vegan* or vegetarian* or nonvegetarian* or non-vegetarian* or paleo* or pescatarian or keto* or mediterranean or meal or meals)
S42	TI (cholesterol or high-cholesterol or HDL or low-cholesterol or LDL or lipoprotein*) OR AB (cholesterol or high-cholesterol or HDL or low-cholesterol or LDL or lipoprotein*)
S41	TI (amino acid* or high-protein* or low-protein* or protein*) OR AB (amino acid* or high-protein* or low-protein* or protein*)
S40	TI (fat or fats or fat-rich or fat-heavy or fatty or high-fat* or low-fat* or omega-3* or omega-6* or oil or oils) OR AB (fat or fats or fat-rich or fat-heavy or fatty or high-fat* or low-fat* or omega-3* or omega-6* or oil or oils)
S39	TI (carbohydrate* or high-carb* or low-carb* or sugar*) OR AB (carbohydrate* or high-carb* or low-carb* or sugar*)
S38	TI (intake or ingest*) OR AB (intake or ingest*)
S37	TI energy OR AB energy
S36	TI (consume# or consuming or consumption) OR AB (consume# or consuming or consumption)
S35	TI (macro-nutrient* or macronutrient* or nutrition* or nutrient* or nutritive*) OR AB (macro-nutrient* or macronutrient* or nutrition* or nutrient* or nutritive*)
S34	TI (calorie* or caloric or high-calorie* or low-calorie* or high-caloric* or low-caloric*) OR AB (calorie* or caloric or high-calorie* or low-calorie* or high-caloric* or low-caloric*)
S33	TI (food* or fed or feed*) OR AB (food* or fed or feed*)
S32	TI (eat or eaten or eats or eating or ate or cook*) OR AB (eat or eaten or eats or eating or ate or cook*)
S31	TI (diet* or supplement*) OR AB (diet* or supplement*)
S30	(MH “Food Security”)
S29	(MH “Food Industry+”)
S28	(MH “Nutrition Policy+”)
S27	(MH “Sports Nutritional Sciences”) OR (MH “Dietetics”) OR (MH “Research, Dietetics”) OR (MH “Nutrigenomics+”)
S26	(MH “Diet Therapy+”)
S25	(MH “Eating Disorders+”)
S24	(MH “Nutrition Services+”)
S23	(MH “Plants, Edible+”)
S22	(MH “Eating Behavior+”) OR (MH “Drinking Behavior+”)
S21	(MH “Nutritional Physiology”) OR (MH “Nutritive Value+”) OR (MH “Digestive System Physiology+”)
S20	(MH “Food and Beverages+”)
S19	(MH “Nutrition+”)
S18	S12 OR S13 OR S14 OR S15 OR S16 OR S17
S17	TI ((drowsy or drowsiness)) OR AB ((drowsy or drowsiness))
S16	TI ((dream* or REM or “rapid eye movement*” or non-REM or NREM)) OR AB ((dream* or REM or “rapid eye movement*” or non-REM or NREM))
S15	I ((sleep* or slept or somnolen*)) OR AB ((sleep* or slept or somnolen*))
S14	(MH “Sleepiness”)
S13	(MH “Sleep Disorders+”)
S12	(MH “Sleep+”)
S11	S1 OR S2 OR S3 OR S4 OR S5 OR S6 OR S7 OR S8 OR S9 OR S10
S10	TI ((antenatal or gestat* or maternal* or maternity or perinatal or pregnan* or prenatal or surroga*)) OR AB ((antenatal or gestat* or maternal* or maternity or perinatal or pregnan* or prenatal or surroga*))
S9	(MH “Delivery, Obstetric+”)
S8	(MH “Expectant Mothers”) OR (MH “Surrogate Mothers”)
S7	(MH “Pregnancy Complications+”)
S6	(MH “Pregnancy+”)
S5	(MH “Maternal-Child Nursing”) OR (MH “Obstetric Nursing”) OR (MH “Perinatal Nursing”)
S4	(MH “Prenatal Bonding”)
S3	(MH “Maternal Health Services+”)
S2	(MH “Obstetric Care+”)
S1	(MH “Delivery Rooms+”)
